# Stent reconstruction for symptomatic inferior cavoatrial anastomotic stenosis following cardiac transplantation

**DOI:** 10.1186/s42155-026-00656-0

**Published:** 2026-02-14

**Authors:** Mayura P. Umapathy, Jeffrey Forris Beecham Chick, David S. Shin, Matthew Abad-Santos, Eric J. Monroe, Sandeep S. Vaidya, Frederic J. Bertino, Jeffrey E. Keenan, Mina S. Makary

**Affiliations:** 1 Division of Vascular and Interventional Radiology, Department of Radiology, The Ohio State University Wexner Medical Center, Columbus, OH 43210 USA; 2 Section of Vascular and Interventional Radiology, Department of Radiology, University of Washington, 1959 Northeast Pacific Street, Seattle, WA 98195 USA; 3 Section of Vascular and Interventional Radiology, Department of Radiology, University of Wisconsin, 600 Highland Avenue, Madison, WI 53792 USA; 4 Division of Interventional Radiology and Image-Guided Medicine, Department of Radiology and Imaging Sciences, Emory University School of Medicine, 1405 Clifton Road, Atlanta, GA 30322 USA; 5 Division of Cardiothoracic Surgery, Department of Surgery, University of Washington, 1959 Northeast Pacific Street, Seattle, WA 98195 USA

To the Editor:

Inferior cavoatrial anastomotic stenosis is a rare complication of orthotopic heart transplantation (OHT) that can cause graft dysfunction and venous congestion. Although multiple surgical and endovascular strategies exist, optimal management remains unclear [1–4]. We present four cases of symptomatic cavoatrial stenosis after OHT treated with dedicated venous stents. All procedures were performed under general anesthesia with initiation of aspirin 81 mg daily post-procedure. Bare-metal, uncovered stents were sized to match the native IVC diameter. When a single stent was insufficient, parallel stents were deployed in a double-barrel configuration to approximate the vessel caliber.

## Case 1

**Fig. 1:**
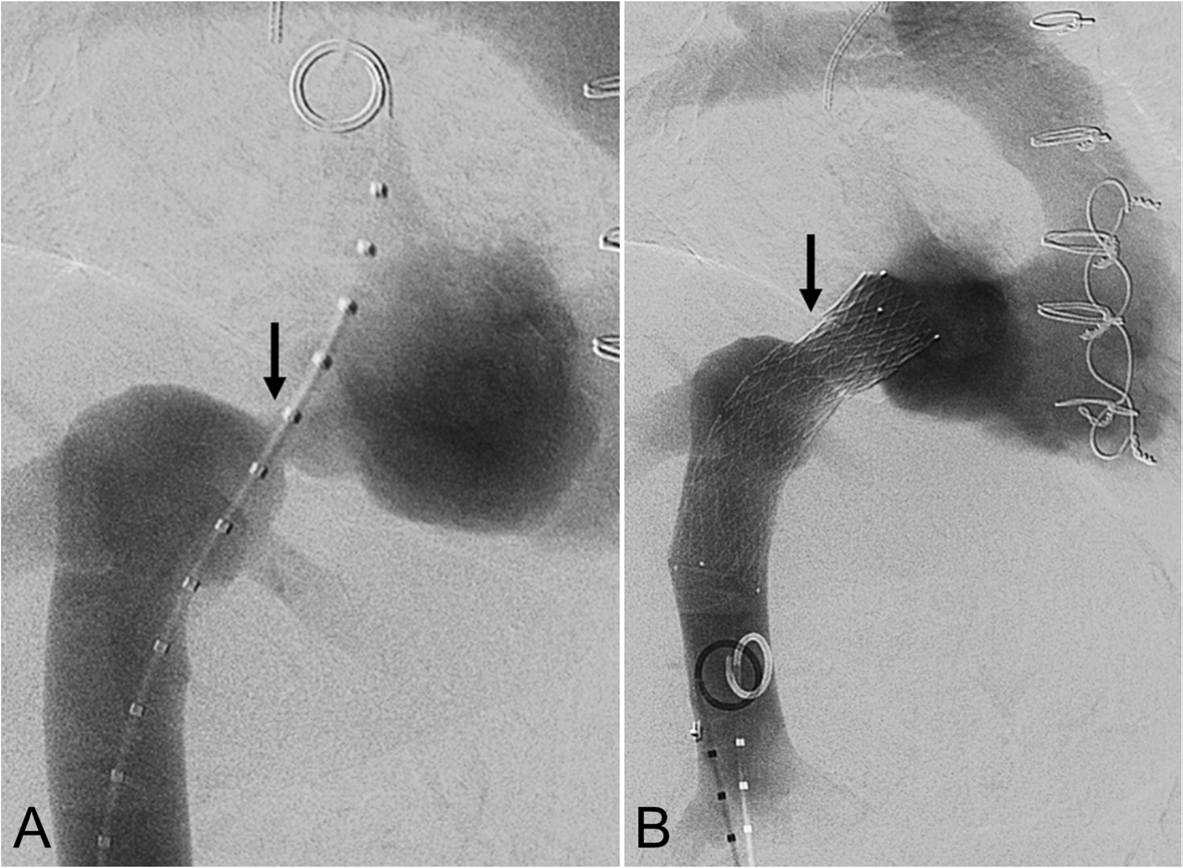
A 68-year-old woman status post OHT 27 days prior for dilated ischemic cardiomyopathy presented with medically refractory bilateral lower extremity edema. **A** Inferior vena cavography demonstrates > 75% anastomotic stenosis (black arrow). **B** Post-reconstruction venography shows the 20-mm × 80-mm Venovo stent (black arrow) in place with resolution of the severe stenosis

A 68-year-old woman, 27 days post-OHT for dilated ischemic cardiomyopathy (ICM), presented with refractory bilateral leg edema. Transthoracic echocardiography showed > 85% cavoatrial anastomotic stenosis, and inferior vena cava (IVC) venography confirmed > 75% narrowing (Fig. 1A). A 20-mm × 80-mm Venovo stent (BD Bard; Franklin Lakes, NJ) was deployed across the stenosis and post-dilated with a 16-mm balloon. Completion venography showed brisk IVC-RA flow (Fig. 1B). Intravascular ultrasound (IVUS) confirmed expansion. Edema resolved by 9 days. Computed tomography venography (CTV) at 140 days confirmed stent patency.

## Case 2

**Fig. 2:**
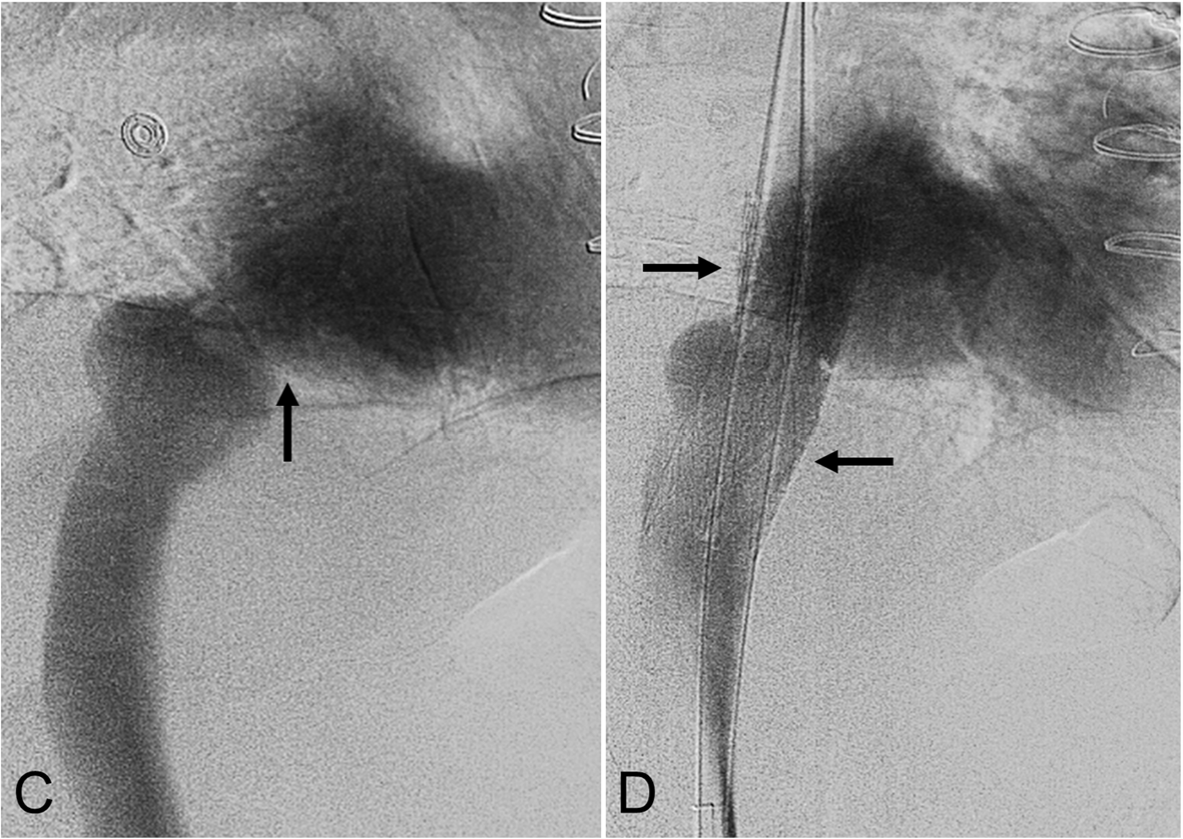
A 69-year-old man status post OHT 167 days prior for ischemic cardiomyopathy presented with dyspnea, bilateral lower extremity edema, and inability to ambulate. **C** Inferior vena cavography demonstrates anastomotic stenosis (black arrow). **D** Post-reconstruction venography shows the two side-by-side 14-mm × 80-mm Abre stents (black arrows) in place without residual stenosis

A 69-year-old man, 167 days post-OHT for ICM, presented with dyspnea and refractory bilateral leg edema. Cardiac catheterization performed 32 days earlier showed loss of IVC respiratory variation. Venography confirmed stenosis with an initial cavoatrial pressure gradient of 5 mmHg (Fig. 2C). Two 14-mm × 80-mm Abre stents (Medtronic; Dublin, Ireland) were deployed side-by-side and dilated to 14 mm. Completion venography demonstrated brisk flow with a 1-mmHg residual gradient (Fig. 2D). Edema decreased substantially at 27-day follow-up and resolved by 106 days. CTV at 125 days confirmed patent stents.

## Case 3

**Fig. 3:**
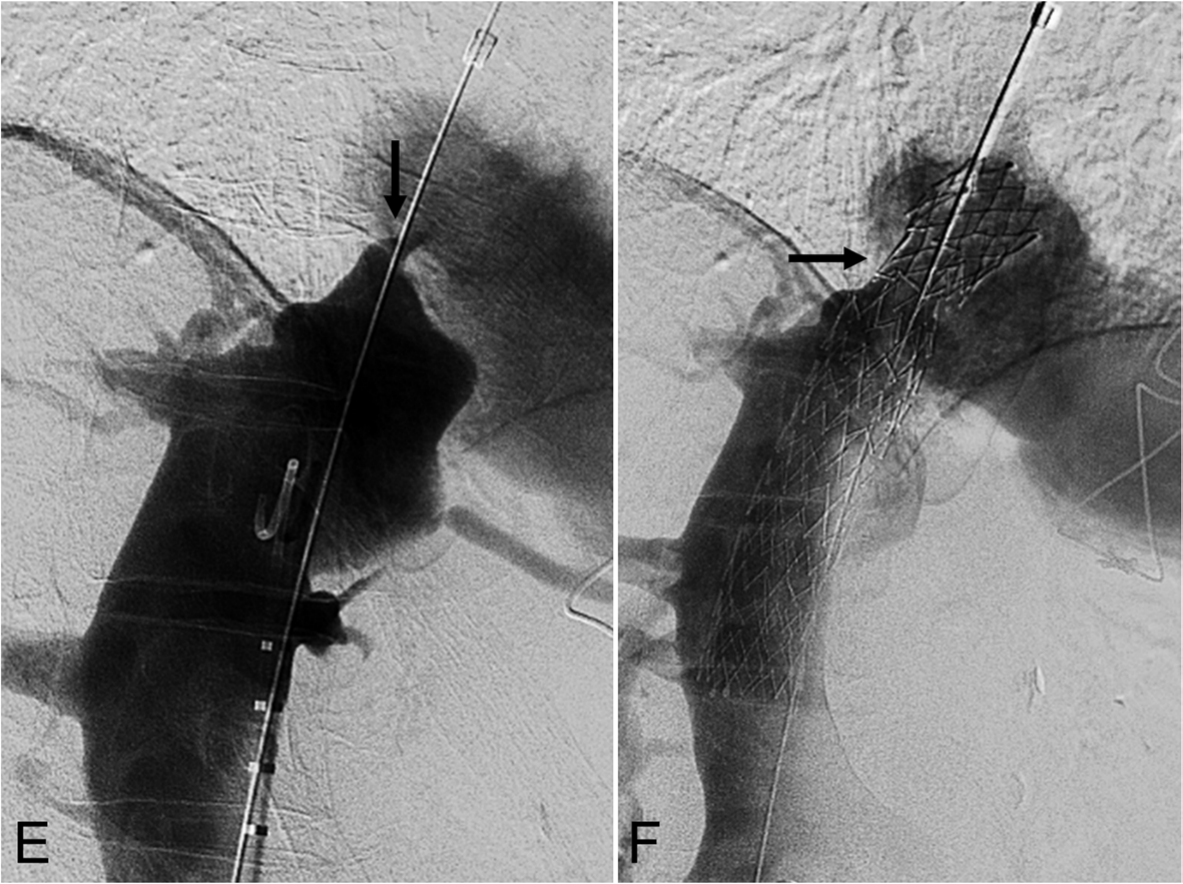
A 76-year-old man status post OHT 4,756 days prior for non-dilated ischemic cardiomyopathy presented with cirrhosis, portal hypertension, and dyspnea on exertion. **E** Inferior vena cavography demonstrates > 95% anastomotic stenosis (black arrow). **F** Post-reconstruction venography shows the 20-mm × 120-mm Abre stent (black arrow) in place with resolution of the near occlusive stenosis

A 76-year-old man, 4,756 days post-OHT for non-dilated ICM, presented with cirrhosis, portal hypertension, and exertional dyspnea. His transplantation required a 20-mm Dacron interposition graft to lengthen the cavoatrial anastomosis. Cardiac catheterization 7 days prior demonstrated an 11-mmHg initial gradient. IVC venography showed > 95% stenosis (Fig. 3E). A 20-mm × 120-mm Abre stent was deployed and post-dilated to 16 mm. Completion venography demonstrated brisk flow. IVUS and cone-beam CT confirmed expansion (Fig. 3F). The final cavoatrial gradient was 2 mmHg. The patient experienced intermittent palpitations post-procedure. Electrocardiography demonstrated normal sinus rhythm. Follow-up imaging showed stable stent position, and symptoms resolved by day 6.

## Case 4

A 78-year-old woman, 1,921 days post-OHT for familial dilated cardiomyopathy, presented with refractory bilateral leg edema. CTV showed cavoatrial kinking, and cardiac catheterization demonstrated a 7-mmHg gradient. Two side-by-side 12-mm × 60-mm Abre stents were deployed and dilated to 12 mm. The final cavoatrial gradient was 1 mmHg. Edema improved by day 79, and CTV at day 111 confirmed stent patency.

OHT patients with cavoatrial stenosis may present with edema, ascites, or hepatic dysfunction, and untreated obstruction may progress to thrombosis. Prior strategies include balloon angioplasty, endovascular stents, and surgical repair [1–4]. Reported stents include Wallstents (Boston Scientific; Marlborough, MA), WallFlex (Boston Scientific), and Gianturco Z-stents (Cook Medical; Bloomington, IN), although these were not designed for large venous reconstruction and may exhibit foreshortening, limited conformability, or instability at the cavoatrial junction, where migration has been reported [1–4].

Dedicated venous stents, including Abre and Venovo, provide minimal foreshortening and improved wall apposition. This case series demonstrates their feasibility and efficacy for symptomatic cavoatrial anastomotic stenosis. Further studies with larger cohorts and longer follow-up are warranted.

## Data Availability

The datasets used and/or analyzed during the current study are available from the corresponding author on reasonable request.

## Abbreviations

OHT: Orthotopic heart transplantation
ICM: Ischemic cardiomyopathy
IVC: Inferior vena cava
IVUS: Intravascular ultrasound
CTV: Computed tomography venography
